# The Relationship between Self-Reported Sitting Time and Vitamin D Levels in Middle-Aged and Elderly Taiwanese Population: A Community-Based Cross-Sectional Study

**DOI:** 10.3390/nu15224766

**Published:** 2023-11-13

**Authors:** Yu-Hsuan Chang, Chun-Ru Lin, Yu-Lin Shih, Chin-Chuan Shih, Jau-Yuan Chen

**Affiliations:** 1 Department of Medical Education, Chang Gung Memorial Hospital, Linkou Branch, Taoyuan City 333, Taiwan; mpq781@cgmh.org.tw (Y.-H.C.);; 2 Department of Family Medicine, Chang Gung Memorial Hospital, Linkou Branch, Taoyuan City 333, Taiwan; elis@cgmh.org.tw; 3 General Administrative Department, United Safety Medical Group, New Taipei City 242, Taiwan; 4 College of Medicine, Chang Gung University, Taoyuan City 333, Taiwan

**Keywords:** 25-hydroxy vitamin D, middle-age and elderly, sitting time

## Abstract

(1) Background: Many studies have revealed a relationship between serum 25-hydroxy vitamin D and physical activity. This study aimed to investigate the relationship between self-reported sitting time and serum 25-hydroxy vitamin D levels in middle-aged and elderly adults in Taiwan. (2) Methods: A total of 396 people were enrolled in our study during a community health examination in Taiwan in 2019. We grouped participants from low to high according to their tertile of serum 25-hydroxy vitamin D levels, using the following categories: deficiency, insufficiency, and sufficiency. Parameters including self-reported sitting time were analyzed between each group. Pearson correlation coefficients were calculated to explore the relationships of serum 25-hydroxy vitamin D levels with age-adjusted risk factors. A scatter plot demonstrated the relationship between serum 25-hydroxy vitamin D levels and self-reported sitting time. The association between serum 25-hydroxy vitamin D levels and self-reported sitting time was assessed by multivariate linear regression with adjustment for age, sex, waist circumference, low-density lipoprotein, triglycerides, and smoking and drinking status. (3) Results: We analyzed the data from 396 participants. A total of 41.4% of participants were male, and the average age of all participants was 64.91 (±8.80) years. The participants in the high serum 25-hydroxy vitamin D group were more likely to have shorter self-reported sitting time. Serum 25-hydroxy vitamin D was negatively correlated (Pearson’s r) with self-reported sitting time, even after adjustment for age. According to the results of multivariate linear regression, vitamin D levels showed a negative association with self-reported sitting time (β = −0.131, *p* = 0.006) after adjustment for age, sex, waist circumference, low-density lipoprotein, triglycerides, and smoking and drinking status. (4) Conclusions: According to our research, self-reported sitting time was inversely correlated with serum 25-hydroxy vitamin D in middle-aged and elderly people in Taiwan. Meanwhile, longer self-reported sitting time can be an independent risk factor for lower serum 25-hydroxy vitamin D levels.

## 1. Introduction

Vitamin D is a fat-soluble vitamin that is obtained from the diet and is generated in the skin after exposure to ultraviolet B rays [1]. Vitamin D exists in two distinct forms: D2 (ergocalciferol) and D3 (cholecalciferol). The difference lies in the structure of the side chains [2]. Whether vitamin D (including D2 and D3) comes from food or is made by the skin, it is converted to 25-hydroxyvitamin D (25(OH)D) by the liver and then converted to 1,25-dihydroxyvitamin D (1,25(OH)2D) by the kidneys [2,3]. Compared to 1,25-dihydroxyvitamin D, 25-hydroxyvitamin D has a longer half-life, making it a better indicator of vitamin D deficiency [4]. The normal level of serum 25-hydroxyvitamin D is not currently agreed upon, and definitions of vitamin deficiencies use different cut points according to the population or other reasons. The Institute of Medicine (IOM) guidelines state that 20 ng/mL of serum 25-hydroxyvitamin D is adequate for the general population [5]. However, the Endocrine Society Clinical Practice Guideline recommends maintaining blood levels of 25-hydroxyvitamin D above 30 ng/mL [6]. Further research also revealed that vitamin D deficiencies increased the risks of bone health, cancer, cardiovascular disease, diabetes, and autoimmune diseases [7,8]. In children, vitamin D deficiency has implications for calcium and phosphorus absorption and the formation of rickets in bone cartilage [9]. Vitamin D also appears to be associated with fractures in older people [10]. Hip fracture risk was reduced by 26% and nonvertebral fracture risk was reduced by 23% with vitamin D supplementation of 700–800 IU daily [3]. Another meta-analysis study with 2426 participants showed that the risk of falls in older adults was reduced by 19% with daily vitamin D supplementation of 700–1000 IU, thereby reducing the incidence of fractures [11]. In 2011–2022, the global prevalence of vitamin D deficiency (25-hydroxyvitamin D < 30 nmol/L) was approximately 14.1% [12], and it caused a large medical burden.

The way people work has changed with the development of modern technology. Much labor has been replaced by computers and labor-saving machines. Sedentary behavior has also become part of most people’s daily lives [13]. Prolonged sedentary periods are linked to an increased risk of diabetes, cardiovascular disease, and both cardiovascular and all-cause mortality [14,15,16]. A previous meta-analysis study with 1,331,468 participants revealed that sitting for more than 8 h a day increases the risk of all-cause mortality and that sitting for more than 6 h a day increases the risk of cardiovascular disease. The relative risk of all-cause mortality estimate was 1.04 (1.03–1.05) for each hour over 8 h per day, and the relative risk of cardiovascular disease estimate was 1.04 (1.03–1.04) for each hour over 6 h per day [17]. Moreover, a significant increase in sedentary time was found regardless of age after the COVID-19 outbreak [18]. Vitamin D deficiency is also related to sedentary time. The odds ratios of having less than 20 ng/mL 25-hydroxyvitamin D were 2.72 and 3.45 for women who were sedentary for more than 9 h and more than 12 h, respectively [19]. Older groups tend to have a longer period of sedentary time. Vitamin D deficiency is prevalent in nearly 20% of middle-aged and elderly adults in the United States [20].

The disadvantage of sedentary behavior is well known. A systematic review encompassing 27 studies documented positive correlations of sedentary behavior with all-cause mortality, type-2 diabetes, fatal and nonfatal cardiovascular disease, and metabolic syndrome [21]. However, few studies have explored the relationship between vitamin D levels and sitting time.

In the past, we made a database aimed at understanding the long-term physical and mental well-being of middle-aged and elderly adults attending community clinics. This study is expected to contribute significantly to the disease management and prevention of middle-aged and elderly adults receiving primary health care. Several publications have already been published based on this database, and this paper is no exception [22,23,24,25,26,27]. This study aimed to investigate differences in the relationship of vitamin D levels with sitting time between middle-aged and older adult populations in Taiwan.

## 2. Materials and Methods

### 2.1. Research Protocol and Search Question

This was a cross-sectional and community-based research study. A broad health survey project was conducted, and participants were recruited in the northern region of Taiwan in 2019. To assure the appropriateness of the participant sample, strict inclusion and exclusion criteria were applied. Considering that older individuals might be unable to participate in our physical fitness assessment, we limited our study to participants up to the age of 85. We included participants who fell within the age range of 50–85 years, resided in the community, had the ability to effectively complete an extensive questionnaire, and demonstrated the ability to go to the clinic on their own. Conversely, we excluded people who had recent cardiovascular disease within the previous three months and did not have complete data. In total, 396 participants were successfully recruited and identified as eligible for further analysis. The sample size determination was based on G*power 3.1 software (Heinrich-Heine-Universität Düsseldorf, Düsseldorf, Germany). A sample size of 296 achieved 90% power using an odds ratio of 2.0, probability of null hypothesis of 0.15, two-tailed alpha criterion of 0.05, and power of 0.9. A total of 396 subjects comprised the sample size of this study, which implied sufficient statistical power [28]. During the course of the health survey, each participant completed a comprehensive questionnaire that included a relevant medical history and personal information. Blood and urine samples were also taken from every participant to measure key biochemical data. Depending on the 25-hydroxyvitamin D levels, the cohort of 396 participants was divided into three different groups. Before enrollment, informed consent was secured from all participants. Ethical approval for this study was granted by the Institutional Review Board of Linkou Chang Gung Memorial Hospital (Approval No. 201801803B0).

### 2.2. Data Collection and Measurements

Data collection involved a questionnaire designed to collect key information, including age, sex, alcohol consumption habits (for example, frequency of drinking more than two days per week), and current smoking status (for example, active smoker or never smoker). Participants provided self-reported data on their alcohol consumption and smoking status. Participants were also asked to self-report how long they sat. Our questionnaire collected data on the average daily sitting time during the last week, focusing on weekdays (excluding sleep time). The measurement unit used for inquiries was minutes per day, which was subsequently converted into hours for statistical analysis. Information on prevalent medical conditions such as metabolic syndrome, hypertension (HTN), diabetes mellitus (DM), dyslipidemia, and chronic kidney disease (CKD) was obtained through a review of medical records. Weight in kilograms was divided by the square of height in meters to calculate body mass index (BMI). Systolic blood pressure (SBP) and diastolic blood pressure (DBP), measured in millimeters of mercury (mmHg), were recorded on several occasions after a period of rest. Waist circumference was measured midway between the iliac crest and the last rib in a horizontal plane while the participant was standing. In the Roche model laboratory at the Taiwan E&Q Clinical Laboratory Biochemical, analyses of participants’ samples were carried out using the Roche cobas^®^ Connecting Module (CCM). The Cobas 8000 is a large, fully automated, random-access biochemical analyzer produced by the company Roche (Basel, Switzerland). Laboratory data included measurements of 25-hydroxyvitamin D (ng/mL), creatinine (mg/dL), triglycerides (TG, mg/dL), low-density lipoprotein (LDL-C, mg/dL), and high-density lipoprotein (HDL-C, mg/dL). For these biochemical specimens, we collected and analyzed them on the same day. After centrifugation, serum samples did not have any additional additives but contained coagulants. The final analysis was performed using the Roche Cobas 8000 module, which includes a biochemical analyzer (C702) and an immunoassay analyzer (E801). Each serum specimen for biochemical and immunoassay testing was refrigerated at 2–8 °C for 7 days to facilitate rechecking.

### 2.3. Assessment of Systemic Disease and Other Variables

Dyslipidemia was defined as achieving one or more of the following: LDL-C level ≥ 130 mg/dL, HDL-C level < 40 mg/dL in men or <50 mg/dL in women, TG level ≥ 150 mg/dL, TC level ≥ 200 mg/dL, or use of lipid-lowering medication [29]. DM was defined as achieving one or more of the following: fasting plasma glucose ≥ 126 mg/dL, documented history of diabetes mellitus, ongoing insulin therapy, or using oral hypoglycemic agents [30]. CKD was defined as the presence of kidney injury as indicated by a urinary albumin-creatinine ratio (ACR) of 30 mg/g or greater or impaired renal function with an estimated glomerular filtration rate (eGFR) of less than 60 mL/min/1.73 m^2^ [31]. HTN was defined as achieving one or more of the following criteria: SBP ≥ 140 mm Hg, DBP ≥ 90 mm Hg, or use of antihypertensive medication to treat hypertension [32]. A regular drinking status was defined as consuming alcohol at least 3 days per week. Regular exercise was defined as exercising at least 2 days per week. According to the Endocrine Society Clinical Practice Guideline, vitamin D deficiency was defined as a 25-hydroxyvitamin D below 20 ng/mL, and vitamin D insufficiency was defined as a 25-hydroxyvitamin D below 30 ng/mL yet not accomplished vitamin D deficiency [6].

### 2.4. Statistical Analysis

The study participants were divided into three different groups based on their 25-hydroxyvitamin D levels: deficiency, insufficiency, and sufficiency. Specifically, 25-hydroxyvitamin D levels < 20, between 20 and 30 (including 20 and 30), and >30 were classified as deficiency, insufficiency, and sufficiency 25-hydroxyvitamin D groups, respectively. Continuous variables, namely, sitting time, age, SBP, DBP, BMI, WC, creatinine, HDL-C, LDL-C, TC, TG, and 25-hydroxyvitamin D, were reported as the means with their respective standard deviations (SDs). Categorical variables such as alcohol consumption, current smoking status, presence of HTN, DM, CKD, sex, and hyperlipidemia are presented as frequencies and percentages. Statistically significant differences were assessed using one-way analysis of variance (ANOVA) for normally distributed data. Pearson correlation coefficients were calculated to evaluate the associations between 25-hydroxyvitamin D levels and other variables, including sitting time, age, TG, HDL-C, LDL-C, BMI, and SBP, with or without adjustment for the factor of age. In addition, linear regression analysis was conducted to investigate the relationship between sitting time and 25-hydroxyvitamin D levels, with sitting time as the dependent variable. Multiple linear regression analysis and multivariate logistic regression analysis were conducted, with adjustments made for variables including age, sex, waist circumference, LDL-C, TG, smoking habits, and alcohol consumption. The threshold for statistical significance was set at a *p* value of less than 0.05. All statistical analyses were conducted with IBM SPSS Statistics version 20.0 for Windows (IBM Corp., Armonk, NY, USA).

## 3. Results

Table 1 shows the characteristics of the study participants. A total of 396 individuals were enrolled. In total, 64 were classified as 25-hydroxyvitamin D deficient, 195 as 25-hydroxyvitamin D insufficient, and 137 as 25-hydroxyvitamin D sufficient. The mean age of the participants was 64.91 ± 8.80 years. The mean sitting time was 4.83 ± 2.66 h per day. The mean 25-hydroxyvitamin D level was 28.32 ± 9.38 ng/mL. No significant differences were observed in creatinine (*p* = 0.102), TG (*p* = 0.372), LDL-C (*p* = 0.348), HDL-C (*p* = 0.458), WC (*p* = 0.544), SBP (*p* = 0.716), DBP (*p* = 0.963), BMI (*p* = 0. 236), current smoking status (*p* = 0.136), alcohol consumption (*p* = 0.022), HTN (*p* = 0.051), DM (*p* = 0.701), dyslipidemia (*p* = 0.741), and CKD (*p* = 0.150) when considering groups based on 25-hydroxyvitamin D levels. However, statistically significant differences were observed for age (*p* = 0.020), sitting time (*p* < 0.001), 25-hydroxyvitamin D (*p* < 0.001), and sex (*p* < 0.001).

**Figure 1:**
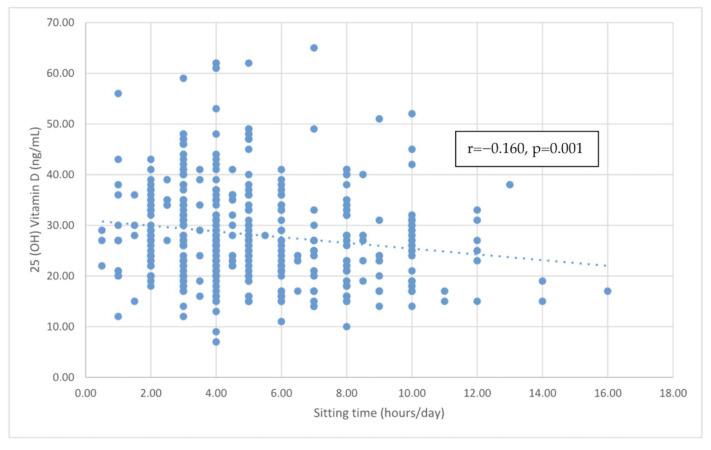
The correlation between sitting time and vitamin D level.

Table 2 reports the correlations between 25-hydroxyvitamin D and various indicators of cardiometabolic risk. A significant negative correlation was found between 25-hydroxyvitamin D and sitting time (r = −0.160, *p* < 0.001), and the linear relationship is reported in Figure 1. However, no significant correlations were found between 25-hydroxyvitamin D and other factors, including age, TG, HDL-C, LDL-C, BMI, and SBP. Even after adjusting for age, the negative correlation between 25-hydroxyvitamin D levels and sitting time remained significant (r = −0.155, *p* = 0.002). In addition, no significant correlations were found between 25-hydroxyvitamin D and the above factors, including TG, HDL-C, LDL-C, BMI, and SBP, after adjusting for age.

Table 3 reports the results of three linear regression models used to explore the association between 25-hydroxyvitamin D levels and sitting time. Model 1 was the unadjusted model, while Model 2 was adjusted for age, sex, WC, LDL-C, and TG. Model 3 included additional adjustments for smoking and alcohol consumption. In all three models, the regression coefficients indicated a significant negative association between 25-hydroxyvitamin D levels and sitting time. The beta coefficient in Model 1 was −0.129 (*p* = 0.008), that in Model 2 was −0.128 (*p* = 0.008), and that in Model 3 was −0.131 (*p* = 0.006). These results suggested that less sitting time was associated with higher levels of 25-hydroxyvitamin D, even after adjusting for various covariates. Furthermore, in Model 3, the unstandardized coefficient is −0.464, indicating that for every additional hour of sitting, the serum 25-hydroxyvitamin D level decreases by 0.464 ng/mL.

We used the same multivariate factor in Table 3 to evaluate the relationship between the sitting time level and vitamin D deficiency (25-hydroxyvitamin D levels < 20) in Table 4. The relationships still remained significantly positive, with an odds ratio of 1.221 in model 1, 1.223 in model 2, and 1.230 in model 3. The result in Model 3 indicates that an additional hour of daily sitting time is associated with a 1.23-fold increased risk of vitamin D deficiency.

## 4. Discussion

In our study, we found a significant negative correlation between 25-hydroxyvitamin D levels and sitting time (r = −0.155, *p* = 0.002) after adjusting for age. However, no significant associations were found between 25-hydroxyvitamin D and other factors, including TG, HDL-C, LDL-C, BMI, and SBP, after adjusting for age. The multiple linear regression models adjusting for age, sex, WC, LDL-C, TG, and smoking and alcohol consumption showed an unstandardized coefficient of −0.464 (*p* = 0.006). The multivariate logistic regression models adjusting for age, sex, WC, LDL-C, TG, and smoking and alcohol consumption showed the odds ratio between the sitting time level and vitamin D deficiency is 1.230 (<0.001). These results imply that long sitting time is an independent risk factor for vitamin deficiency, even when adjusting for multiple confounding factors.

Vitamin D deficiency is a common health problem worldwide, especially among older people. In a northern Taiwan community-based cohort study, vitamin D deficiencies were most common in people aged 30–39 (38.4%), with a gradual decline after age 40. However, the prevalence increases from the age of eighty. The prevalence of vitamin D deficiency reached 7.2% between 70 and 79 years of age and became 12.4% after the age of 80 years old [33]. The highest prevalence of vitamin D deficiency in the 30–39 age group may be because they have a higher average level of education or indoor work and pay more attention to skin whitening and sun protection [33,34]. Vitamin D deficiency occurs in the elderly population due to reduced levels of 7-dehydrocholesterol in the epidermis and reduced kidney function, which reduces vitamin D production [35].

We also investigated the relationship between 25-hydroxyvitamin D and various indicators of cardiometabolic parameters. According to the Nutrition and Health Survey in Taiwan, the incidence of metabolic syndrome surged from 13.6% to 25.5% between the 1993–1996 and 2005–2008 surveys [36]. Metabolic syndrome affects more than 20% of the adult population in the Asia–Pacific region, with prevalence increasing over the long term [37]. There was also an increase with age in the prevalence of metabolic syndrome. A meta-analysis in mainland China showed that the prevalence rates of metabolic syndrome in groups aged 15–39, 40–59, and ≥60 years were 13.9%, 26.4%, and 32.4%, respectively [38]. Therefore, these cardiometabolic factors are important in Taiwan, especially for the middle-aged and elderly populations. We found no significant correlations between 25-hydroxyvitamin D and other factors, including TG, HDL-C, LDL-C, BMI, and SBP. Previous studies and results showed that 25-hydroxyvitamin D was not associated with weight, BMI, WC, SBP, DBP, or HDL-C, which is similar to our results [39]. However, previous studies and results showed that 25-hydroxyvitamin D was associated with LDL-C. The mechanisms through which vitamin D may influence lipid profiles are not clear but may include inhibition of PTH secretion, increase in calcium levels to influence blood fats, and influence on insulin release and sensitivity [40,41,42]. The results of this study are different from those of previous studies, which may be due to the different races of the participants. This is important for elucidating the relationships of 25-hydroxyvitamin D with other factors, especially the prevalence of metabolic syndrome in Taiwan, which is high and on the rise.

After adjustment for factors including age, sex, WC, LDL-C, TG, and smoking and alcohol consumption, the regression coefficients indicated a significant negative association between 25-hydroxyvitamin D levels and sitting time. There are only a few studies which have investigated the association between 25-hydroxyvitamin D levels and sitting time [43,44]. One of the papers, from Chile, investigated the association between different patterns of physical activity and sedentary time and vitamin D deficiency (<12 ng/mL). The results of the study found similar results to our paper in the adult group [43]. However, there are also some differences between them. First, our study participants were from the northern region of Taiwan and were over the age of 50 years, while those from the above study were from Chile and were limited to women over the age of 18 years. Furthermore, we used different questionnaires to record sedentary time. In addition, lifestyle differences lead to different prevalences of vitamin D deficiency, revealing that older people were more likely to have vitamin D deficiency (<20 ng/mL) than younger people, with prevalence rates of 64.9% and 51.6%, respectively. The prevalence in each group was obviously higher than the prevalence (22.4%) of individuals in Taiwan [33]. This may be because Taiwan has more people supplementing with vitamin D and regularly undergoing health exams. Last, the odds ratios for vitamin D deficiency in the long (>8 h) and medium (4–8 h) sedentary groups did not significantly differ from the short (<4 h) sedentary group in elderly individuals > 65 y. Differences in study design, population, statistical methods, etc., may explain the differences in our results. However, statistically significant differences could be found with different cutoff points (<14 ng/mL) or age groups. The results of the previous study showed that the odds ratios for vitamin D deficiency (<20 ng/mL) in the long (>8 h) and medium (4–8 h) sedentary groups compared with the short (<4 h) sedentary group in adults aged 18–64 years were 2.1 and 1.7, respectively. These previous studies can still be used as supporting evidence to corroborate the results of our research. The mechanisms underlying the relationship between sitting time and vitamin D deficiency are not yet fully understood. The most common hypothesis for this is that less time spent sitting leads to more exposure to the sun, which leads to the production of vitamin D [44]. Another hypothesis is that physical inactivity and obesity are linked; vitamin D is fat-soluble and is easily stored in adipose tissue, resulting in changes in serum levels of vitamin D [45].

In 2011–2022, the global prevalence of vitamin D deficiency (25-hydroxyvitamin D < 30 nmol/L) was approximately 14.1% [12], which resulted in a large medical burden. In a community sample in northern Taiwan, vitamin D insufficiency (25-hydroxyvitamin D levels < 20 ng/L) was 22.4% prevalent [33]. A recent study revealed that vitamin D supplements prevent nearly 30,000 cancer-related deaths in individuals over 50 years of age, saving a total of 254 million Euros annually in Germany [46]. Taiwan turned into an aged society in 1993 and then an aging society in April 2018, when the number of elderly people increased to 14.10% of the total citizens [47]. Greater attention to older people’s health issues is warranted. This information provides an understanding of the importance of vitamin D, not only in terms of health but also in terms of medical resources, and a reflection on the issue of vitamin D deficiency in the Taiwanese population in elderly people.

Sedentary time is associated with an increased risk of diabetes, cardiovascular disease, and cardiovascular and all-cause mortality [14,15,16]. One meta-analysis compared the differences in disease and mortality risk between the highest and lowest classifications of sedentary time. The higher group was associated with a 112 percent higher risk of diabetes, 147 percent higher risk of cardiovascular disease, 90 percent higher risk of cardiovascular mortality, and 49 percent higher risk of all-cause mortality [14]. The health risks of sedentary time are beginning to receive attention. Over the past decade, the quantity of publications addressing sedentary time has grown by a factor of 15. Sedentary time is also related to age. Time spent sitting increases with age for children and teenagers. However, in adults, the findings on the association between age and sitting time are complicated. In older adults, older age was often associated with more sedentary time [15]. Moreover, COVID-19 has garnered considerable attention in the field of sedentary time research as billions of people were asked to stay at home in 2020 [48]. The average sitting time for adults in the COVID-19 group was 510.5 ± 167.9 min per day, and for the elderly group, it was 586.3 ± 25.2 min per day. The former increased by 126.9 ± 42.2 min per day, and the latter increased by 46.9 ± 22.0 min [18].

Our study has some limitations. First, the generalizability of our findings to other populations may be limited because the participants in our study were recruited from northern Taiwan. Second, the sample size in our study was relatively small. Third, the cross-sectional design of our study limits our ability to establish a causal relationship between sitting time and 25-hydroxyvitamin D. In addition, daily physical activity was not recorded, which may affect the results of the analysis. Future studies may use accelerometers to measure physical activity intensity, duration, and sedentary behavior to provide more accurate and reliable data. In addition, accelerometers may better capture sedentary time than self-report measures as there is often a discrepancy between self-reported sedentary time and actual sedentary behavior. The inclusion of personal electronic devices with accelerometers in future studies is a priority. Further meta-analyses, randomized controlled trials, and prospective cohort studies are needed to validate and consolidate our findings.

## 5. Conclusions

This study informed that self-reported sitting time had a negative relation with serum 25-hydroxy vitamin D in middle-aged and elderly people in Taiwan. Meanwhile, longer self-reported sitting time can be an independent risk factor of lower serum 25-hydroxy vitamin D levels. To confirm our findings, further prospective cohort studies are needed.

## Figures and Tables

**Table 1:** Demographic characteristics of participants according to 25-hydroxyvitamin D level.

25-Hydroxyvitamin D					
	Total	Deficiency (<20)	Insufficiency (20–30)	Sufficiency (>30)	
Variable	*n* = 396	*n* = 64	*n* = 195	*n* = 137	*p* Value
Sitting time (hours/day)	4.83 ± 2.66	6.34 ± 3.30	4.57 ± 2.40	4.51 ± 2.46	<0.001
25 (OH) Vitamin D (ng/mL)	28.32 ± 9.38	16.14 ± 2.57	25.15 ± 3.06	38.53 ± 7.09	<0.001
Age (year)	64.91 ± 8.80	62.17 ± 8.58	65.55 ± 8.98	65.26 ± 8.47	0.020
Creatinine (mg/dl)	0.87 ± 0.43	0.82 ± 0.31	0.84 ± 0.56	0.93 ± 0.22	0.102
Triglyceride (mg/dl)	141.07 ± 110.00	145.77 ± 119.08	146.69 ± 131.35	130.88 ± 61.71	0.372
HDL-C (mg/dl)	53.46 ± 14.48	53.98 ± 13.26	54.07 ± 14.39	52.35 ± 15.18	0.458
LDL-C (mg/dl)	109.69 ± 33.99	113.02 ± 37.08	109.66 ± 32.78	108.18 ± 34.34	0.348
WC (cm)	85.36 ± 10.83	84.55 ± 12.41	85.49 ± 11.01	85.54 ± 9.80	0.544
SBP (mmHg)	137.30 ± 17.49	137.39 ± 17.07	136.53 ± 17.15	138.36 ± 18.22	0.716
DBP (mmHg)	85.19 ± 10.98	85.94 ± 11.07	84.36 ± 10.81	86.01 ± 11.17	0.963
BMI (kg/m^2^)	25.59 ± 3.84	25.87 ± 5.15	25.80 ± 3.66	25.18 ± 3.34	0.236
Gender, male (%)	164 (41.4%)	14 (21.9%)	70 (35.9%)	80 (58.4%)	<0.001
smoking (%)	50 (12.6%)	5 (7.8%)	24 (12.3%)	21 (15.3%)	0.136
drinking (%)	28 (7.1%)	3 (4.7%)	10 (5.1%)	15 (10.9%)	0.052
HTN (%)	201 (50.9%)	30 (46.9%)	91 (46.7%)	80 (58.4%)	0.051
DM (%)	133 (33.6%)	19 (29.7%)	68 (34.9%)	46 (33.6%)	0.701
Dyslipidemia (%)	153 (38.6%)	24 (37.5%)	79 (40.5%)	50 (36.5%)	0.741
CKD (%)	100 (25.3%)	16 (25.0%)	41 (21.0%)	43 (31.4%)	0.150

Note: Data expressed as mean ± SD for continuous variables and *n* (%) for categorical variables. Abbreviations: HDL-C = high density lipoprotein; LDL-C = low density lipoprotein; WC = waist circumference; SBP = systolic blood pressure; DBP = diastolic blood pressure; BMI = body mass index; HTN = hypertension; DM = diabetes mellitus; CKD = chronic kidney disease.

**Table 2:** The correlation between vitamin D level and cardiometabolic risk factors.

25-Hydroxyvitamin D				
	Unadjusted		Adjusted for Age	
Variables	Correlation	*p*-Value	Correlation	*p*-Value
Sitting time (hours/day)	−0.160	<0.001	−0.155	0.002
Age (year)	0.096	0.056	NA	NA
Triglyceride (mg/dl)	−0.065	0.199	−0.056	0.263
HDL-C(mg/dl)	−0.026	0.610	−0.029	0.564
LDL-C (mg/dl)	−0.034	0.495	−0.014	0.774
BMI (kg/m^2^)	−0.084	0.097	−0.078	0.120
SBP (mmHg)	0.015	0.770	−0.006	0.901

Abbreviations: HDL-C—high density lipoprotein; LDL-C—low density lipoprotein; SBP = systolic blood pressure; BMI = body mass index.

**Table 3:** Multiple linear regression analysis for vitamin D levels in relation to sitting time after adjustment for potential confounders.

		Model 1				Model 2				Model 3		
	B	SE	β	*p* Value	B	SE	β	*p* Value	B	SE	β	*p* Value
sitting time	−0.455	0.171	−0.129	0.008	−0.450	0.170	−0.128	0.008	−0.464	0.169	−0.131	0.006

Abbreviations: B, unstandardized coefficients; S.E., standard error; β, standardized coefficients. Model 1 was adjusted for age and gender; Model 2 was adjusted for age, gender, waist circumference, low density lipoprotein, and triglyceride; Model 3 was adjusted for age, gender, waist circumference, low density lipoprotein, triglyceride, smoking, and drinking.

**Table 4:** Multivariate logistic regression analysis of the association between sitting time level and vitamin D deficiency (25-hydroxyvitamin D levels < 20) after adjustment for potential confounders.

	Model 1			Model 2			Model 3		
Variable	OR	95% CI	*p*-Value	OR	95% CI	*p*-Value	OR	95% CI	*p*-Value
Sitting time	1.221	1.109 to 1.344	<0.001	1.223	1.109 to 1.347	<0.001	1.230	1.114 to 1.358	<0.001

Abbreviations: OR, odds ratio; 95% CI, 95% confidence interval. Model 1 was adjusted for age and gender; Model 2 was adjusted for age, gender, waist circumference, low density lipoprotein, and triglyceride; Model 3 was adjusted for age, gender, waist circumference, low density lipoprotein, triglyceride, smoking, and drinking.

## Data Availability

The raw data supporting the conclusions of the article will be made available by the authors without undue reservation.
